# Creating a Collaborative Platform for the Development of Community Interventions to Prevent Non-Communicable Diseases

**DOI:** 10.3390/ijerph16050676

**Published:** 2019-02-26

**Authors:** Sebastian del Busto, Inés Galindo, Juan Jesús Hernandez, Francisco Camarelles, Esther Nieto, Águeda Caballero, María Sandín Vázquez

**Affiliations:** 1 Prevention and Health Promotion programs coordinator, Spanish Association Against Cancer (AECC), 28010 Madrid, Spain; sebastian.delbusto@aecc.es; 2 Management Director, Spanish Heart Foundation (FEC), 28028 Madrid, Spain; igalindo@fundaciondelcorazon.com; 3 Health Department, Spanish Red Cross, 28003 Madrid, Spain; jjhernandez@cruzroja.es; 4 President of Preventive Activities and Health Promotion Program (PAPPS), Spanish Society of Family and Community Medicine (semFYC), Family Doctor, 28004 Madrid, Spain; fcamarelles@telefonica.net; 5 General Secretariat, Spanish Federation of Community Nursing Associations (FAECAP), 28008 Madrid, Spain; secretaria@faecap.com; 6 Lifestyle working group, Spanish Diabetes Society (SED), 28002 Madrid, Spain; caballero.figueroa@gmail.com; 7 Surgery and Medical and Social Sciences Department, School of Medicine, University of Alcalá de Henares, 28805 Madrid, Spain

**Keywords:** community health, chronic disease, prevention, partnerships, health promotion, structural interventions, social determinants, multi-component interventions, NCD

## Abstract

Chronic diseases, also known as non-communicable diseases (NCD) are one of the most important public health problems of our time. Many of these diseases can be reduced by achieving healthy lifestyles. Community interventions are very useful in reducing these types of diseases since they have a direct impact over daily conditions and are adjustable to the complex situations that they carry. This article describes the process of the creation of a collaborative platform for the design and implementation of community interventions to prevent NCDs. This platform includes six non-governmental organizations who have aligned their prevention and health promotion objectives to develop joint community interventions. The intervention levels approach, based on the socio-ecological model has been the basic model to structure the working groups of the platform. Dealing with institutional differences, complexity and variability of contexts, defining the roles and responsibilities and managing the resources are key elements to have in mind to achieve good relations and functional partnerships to design and implement effective community interventions at different levels. Institutional recognition, support and planning based on local priorities are also key elements for these kinds of platforms to be successful, sustainable and, therefore, have an impact on people’s health.

## 1. Introduction

### 1.1. Non-Communicable Diseases and Community Interventions

In 2014, 92% of the deaths in Spain were caused by non-communicable diseases (heart disease, cancer, chronic respiratory disease, diabetes, among others) [1] and 80% of the public health expenditure was dedicated to chronic diseases [2]. According to the World Health Organization (WHO), intervening over risk factors can prevent up to 80% of premature heart disease and diabetes cases [3] and from 30 to 50% of cancer cases in general [4].

The very complex nature of these types of diseases and their multifactorial origin make necessary the implementation of complex strategies. The most effective public health interventions are made up from an evidence-based technical package that combines different actions at different levels that together will enhance, achieving notorious improvements over a risk behavior or a punctual disease [5].

Accordingly, community interventions are fundamental elements in health promotion and NCD prevention. Their efficacy has been proven in different ages [6,7], in different settings of the community [8], in different countries [9] and in different risk factors, like increasing the consumption of fruits and vegetables or decreasing obesity rates [10,11], up to improving people’s participation in vaccination programs [12].

In addition, well-designed community interventions by local governments with community participation can also be effective in reducing health inequalities [13]. In Spain, community interventions are seen as fundamental elements to support public health policies that improve people’s health by influencing social determinants of health taking in account equity, citizen participation and intersectoral work as transversal matters [14]. Much more needs to be done, especially in regards of coordination, as exposed below.

### 1.2. Articulating Non-Governmental Institutions

Collaborative work between different institutions and sectors is fundamental to the implementation of any successful public health intervention [5] and is critical in any strategy that focuses on reducing NCDs. It is so important, that it is one of the key elements the WHO urges member states to develop [15]. This is also applicable to designing and implementing community interventions to reduce NCDs [16].

In Spain, the situation of NCD prevention is characterized by being very fragmented and even though there are a lot of good initiatives and programs [17] real coordination between institutions at different levels and territories has not been achieved. Research shows that in other parts of the world there has also been difficulties in consolidating collaborative platforms and partnerships, showing as the most important barriers to budget management, lack of political and institutional support, dealing with the complexity and variability of scenarios and approaches [18] depending on the political situation or sustaining the collaboration in time [19].

In our case, it is the same between non-governmental institutions. Different institutions have great initiatives [20] but it is necessary to improve coordination and to create synergies to optimize resources, increase outreach and impact as part of the greater objective, which is reducing NCDs in Spain.

This paper describes the first part of a project that consists of achieving an institutional partnership to create a collaborative platform for the development of community interventions to prevent NCDs.

### 1.3. Inspiring Principles

The whole initiative was inspired by some basic theoretical principles:

- Social Determinants of Health. The social determinants of health are the conditions in which people are born, grow, live, work and get old, including the health system [21,22]. These conditions are crucial because of their influence on other health determinants (biological factors, health system factors, environmental factors, lifestyle factors). At the community level, all the elements from the environment that can help make the healthy choice the easy choice are especially important. We wanted to extend our approach by creating the right conditions, so our community interventions could influence the social determinants of health.
- The Health Impact Pyramid. Describes the impact of different public health actions and the gradient of personal effort needed in each action. It says for example, that counseling and education requires much more individual effort and has less population impact than changing the context to make default decisions healthy [23]. A greater number of non-governmental institutions have been working for a long time in health education and counseling, looking to inform and raise awareness about different risk factors or diseases. In this sense, refocusing the activities to complement what it´s done with actions that change context should be the way to go.
- Socio-ecological approach—Levels of intervention. The socio-ecological approach explains the interaction between different factors that lead to healthy or unhealthy behavior and decisions. This approach also organizes these factors in different levels [24,25].
  Level 3: Factors determined by governmental structures, production systems, laws and politics.
  Level 2: Factors determined by the conditions in which people´s day to day activities take place. Communities, common environments, workplaces, schools.
  Level 1: Factors determined by social norms, family, educations and the components of personal decision (expectations, motivation, self-efficacy, acceptance).
  Different health promotion initiatives have been structured by this approach [26] and, in this case, the working groups have been structured in the three levels with the intention of working harmonized actions to enhance the impact.
- Proportional Universalism. Proportional universalism, according to Sir Michael Marmot, means that even though health promotion actions must be universal, these actions must follow a scale and intensity that is proportional to the level of disadvantage. Dedicating the best efforts to those with more disadvantage without disregarding the rest [27]. This is a key concept that we had in mind to establish priorities in the design of the interventions.

These basic principles were key elements in the design of the project and the new prevention and health promotion strategy of the Spanish Association Against Cancer (AECC, for its Spanish name), as explained below.

## 2. Materials and Methods

### 2.1. Theoretical Appproach

Working in NCD prevention brings a great deal of possibilities. Different types of actions and a different methodology can be applied, each one of them having different results depending on the characteristics of the target population and the personal effort needed.

For the design of the platform, a group of prevention and health promotion experts from the National Health School, Madrid´s regional and local council, Spanish Network of Healthy Universities and the Spanish Society for the Study of Obesity were invited in April to a Think Tank (a group of experts brought together, usually by a government, to develop ideas on a subject and to make suggestions for action) [28]. The experts were five medical doctors who specialized in different areas related to various topics (e.g., endocrinology, preventive medicine and public health, physical activity and public policies) and one very experienced community nurse. In this think tank, the most relevant aspects about the creation of the platform were discussed considering the basic theoretical principles previously described. 

At the end, the most important conclusions were that we needed to combine all previously explained concepts and structure our interventions strategically, so we could achieve a bigger impact, in this case focusing on the second level of intervention, the community level. We also learned that we needed to understand the complex nature of networks, its ethical implications and key elements, expressed in the literature [29,30], to increase our chances of success. 

### 2.2. Participants and Intervention Design

This initiative starts as part of the redesign of the prevention and health promotion strategy that the Spanish Association Against Cancer (AECC) is working on with the objective of achieving a scalable and sustainable model. The AECC is a private nonprofit association that has been working for over 35 years in cancer prevention and health promotion activities, among others. Most of the prevention actions that the AECC carry out are on schools, reaching more than 200,000 kids last year.

To this effect, in 2017 the prevention concept changed in the institution, going from preventing cancer to promoting healthy habits, from working in education and counseling to thinking about actions that could change the conditions in which people live, and going from working in schools to focusing on working in other settings. The most important change occurred when it was decided to create a collaborative platform based on partnerships with other institutions to achieve a common agenda in the prevention of NCDs.

Being a group of institutions, and not just the AECC, would increase our influence over decision-makers and authorities responsible for many of the most important and permanent decisions needed for the prevention of NCDs. In the same way, this would allow us to be change agents in the way NCD prevention and health promotion is done in Spain, hopefully influencing more and more local networks and platforms from all around the country.

To build the collaborative platform, three work stages were followed:

Stage 1. A partners mapping [31] was done creating a list of institutions working in NCDs prevention at a national level. This mapping was done during January and February of 2018. Different criteria were established to evaluate each institution. The criteria was: Similarity of objectives, economic situation (based on annual budget), influence (based on scientific production, internet blogs, publicity, social media), conflicts of interest (based on fund raising and current partners) and political incidence (based on each institution work results at this level). All criteria had the same importance, a general discussion was held by the AECC team, and consensus was reached when scoring each institution.

Stage 2. According to the evaluation of the potential partners, the most suitable institutions to start the platform with were identified (Table 1). Between April and September of 2018 each institution was contacted establishing meetings and presenting the project. Depending on the feedback of each potential partner and the experts of the Think Tank, a final proposal was presented to the partners.

Stage 3. A meeting of all the founding partners was held in November of 2018 to work in the elaboration of the background technical documents and to draft a terms of reference document (agreement). After negotiating the organizational structure, operative structure, roles, responsibilities, economic sustainability, naming, common agenda and action plan, an agreement was achieved. The most negotiated terms were economic sustainability and responsibilities. The AECC volunteered to act as secretariat assuming this responsibility and the first administrative expenses. Every organization in the platform agreed to assign one professional to each working group. It was not necessary to be part of all three working groups, but every partner decided to do it. With the consent of the partners and the agreement made, the organizational structure and the functional structure (working groups) were defined. 

The platform started to operate in December 2018 (every founding partner agreed to its internal approval) and the formal signature and launch of the platform will be done in a communications event in the first semester of 2019.

## 3. Results

The result of the first part of this project that consists in achieving institutional partnership to create a collaborative platform for the development of community interventions to prevent NCDs is the consolidation of the platform. This platform has an organizational structure and a functional structure.

### 3.1. Organizational Structure

**Figure 1:**
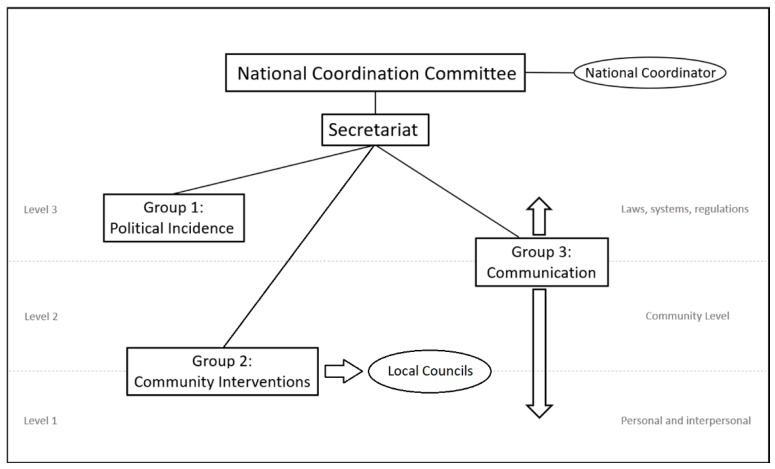
Organizational and operative structure of the collaborative platform. National Coordination Committee manages three working groups. Each group works at a different intervention level. Some groups can work at more than one level.

This collaborative platform for the development of community interventions to NCDs prevention is made up by six non-governmental organizations (founding partners) and is coordinated by a National Coordination Committee (Figure 1). All founding partners are part of this committee through one representative. Each of the funding partners chooses its representative in the committee.

The National Coordination Committee members act as decision-makers and commit to following the platform action plan, previously accepted by all parts. Every decision is made by consensus. The National Coordination Committee also has other functions:

- Makes recommendations, offers guidance and support to the Secretariat since the foundation of the platform about the coordination of the work groups.
- Analyzes and approves the work plan of each group including the evaluation system.
- Works as ambassadors of the initiative, spreading the word and communicating it in all their institutional and professional channels.

This committee will choose by unanimity among its members a National Representative. The National Representative is the top representative of the initiative in external events. The National Coordination Committee receives help from the Secretariat for logistical and administrative tasks. It meets quarterly via videoconference or face-to-face for status meetings. The last meeting of the year is the evaluation meeting and proposal of next year’s action plan. Meetings are organized by the Secretariat. The secretariat communicates with the National Committee members via email or phone.

### 3.2. Functional Structure

Three working groups were created as the functional structure (Figure 1) made up by professionals of every founding institution (Table 1). Each group has a coordinator/representative chosen by the group members by consensus and is the contact person and is responsible for reporting the group activity to the Secretariat and the National Coordination Committee in the quarterly meetings. Each group has been created to work on all three intervention levels with a different methodology, aiming to achieve multi-component interventions [32] that enhance the impact. 

Each working group makes his action plan and reports to the National Coordination Committee in the quarterly meetings. Therefore, all the activity is well followed and supported. The objectives for each working group are:

1. Group 1: Political incidence
  - Influence decision-makers to make permanent decisions and changes that promote healthy habits.
  - Create political structures and networks that support health promotion through community interventions.
2. Group 2: Community Interventions
  This group is the one in charge of working with local councils to:
  - Promote the implementation of the National Prevention and Health Promotion Strategy.
  - Promote the local implementation of the strategy (community interventions).
  - Design and implement community interventions to promote healthy lifestyles (health education and changes in the context).
  - Design and offer toolboxes and resources for the implementation of these local actions.
3. Group 3: Social Marketing and Communication
  - Design health promotion interventions through social marketing and communications.
  - Create an internet repository of all the successful experiences.
  - Perform as communications team for the platform.

The most important points of the process are summarized in the Appendix A (Table A1). In addition, keeping in touch with the Think Tank experts, communicating the progress and asking for feedback is very important to improve the platforms performance.

### 3.3. Developing Community Interventions to Prevent NCDs—National Prevention and Health Promotion Strategy

The local implementation of the National Prevention and Health Promotion Strategy is based on creating local multi-sectorial roundtables. Annually, the Spanish Health Ministry offers economic incentives to those local councils who want to be part of the strategy.

For the development of the platform’s community interventions (Group 2), the idea is to follow the local implementation guideline of the national strategy [33]. This guideline establishes a sequence of actions to design, implement and evaluate community interventions. 

This process can be explained in three parts:

#### 3.3.1. Part 1. Recruiting Local Councils and Disseminating the Initiative

The health promotion team of the Ministry of Health has a list of the local councils that are already part of the strategy (they already have a multi-sectorial round table that designs and implements health promotion community interventions) and those who are in the process of joining. The platforms working group (Group 2) will study this list and evaluate which other local councils could join the strategy. This group will work on elaborating a prioritization tool (proportional universalism) that can help to identify which local councils are more in need. 

At the same time, the group will work on disseminating the strategy and try to increase the knowledge about the economic incentives to get more local councils to join the strategy. We will offer technical support to all local councils that want to join the strategy if needed. For local councils that are already part of the strategy, technical support will also be provided if needed. 

#### 3.3.2. Part 2. Establishing an Inter-Sectorial Round table and Identifying Community Resources

If a local council joins the strategy, we will work with them in two initial steps:

- Creating the multi-sectorial round table. Our community interventions team (group 2) will support local councils in the creation of the round tables and will be a part of these tables. These tables will be led by the local council´s health promotion team.
- Identifying community resources. Health asset mapping [34] is done to get all of the information of health assets of the community (institutions, associations, physical resources or local activities that promote health). Health promotion based on asset mapping helps to introduce concepts like health equity and participation to all the processes [35]. Also, having all the health promotion resources identified helps a great deal in the design and implementation of new health promotion community interventions, whether it is promoting some resources that already exist, or generating new ones.

#### 3.3.3. Part 3. Implementing Specific Community Interventions

The next step after creating the local multi-sectorial round table and doing the health promotion asset mapping is designing and implementing specific community interventions. The National Prevention and Health Promotion Strategy has seven prioritized interventions. These interventions work as successful experiences that are ready to be replicated. Also, in the local multi-sectorial round table new interventions can be designed and implemented. Our team will offer technical support through the whole process. All this is possible due to the strong territorial presence that some of the partners have and years of experience working on prevention programs and health education.

During all the actions, community participation is promoted informing citizens at least once a year and establishing participation mechanisms (social media, public consults, forums, etc.) that allow the local government to hear opinions about the implementation of the strategy and the multi-sectorial round table´s work. In these actions, it is very important that local health promotion professionals get involved as facilitators.

Right now, the platform is starting the process in six Spanish provinces that include Madrid, Guipúzcoa, Valencia, Tenerife, Almería and Álava. After following and evaluating these first initiatives, the local implementation of the national strategy will be evaluated and adjusted to expand the model to other Spanish provinces.

At the time of writing this article, the first implementation actions were being made in the six provinces mentioned above.

### 3.4. The Importance of Sustainability

To guarantee the sustainability of the platform, three aspects have been crucial:

1. Involve Spanish Health Ministry as much as possible. Several meetings have been held with the health promotion team of the Ministry for support on the local implementation of the strategy. Working with a permanent institution such as the Spanish Health Ministry and helping to implement their strategy is fundamental for the platform to not be just another sporadic effort but to remain functioning in time.

2. Training local platform health promotion professionals. A local health online course (elaborated by the Spanish Health Ministry [36]) is offered to all platform professionals that are participating in the first local initiatives of the platform. A health promotion workshop is also being coordinated for all health promotion professionals of the platform. Training our professionals is very important to achieve commitment, motivation and a good technical level for the implementation of the interventions.

3. Creating health promotion permanent structures. Local implementation of the strategy means the creation of local multi-sectorial round tables. These tables are permanent and have a longer duration than the duration any initiative of this type can have.

4. Monitoring and evaluating. For this part of the process (creating a collaborative platform), qualitative evaluations are going to be done to follow the evolution of the implementation focusing on the elements that we have previously established as important: The involvement of the Spanish Health Ministry, training local health promotion professionals and the creation of local and functional multi-sectorial round tables.

## 4. Discussion

We think that creating this platform will change the way NCD prevention and health promotion is done in Spain. Non-governmental institutions, united and well-coordinated, could be a great agent of social mobilization, could contribute to increased awareness about these diseases, could create more pressure over decision-makers (government) and could bring solutions in the shape of well-designed and implemented community interventions.

Different models of institutional collaboration and partnerships have proved to be productive and successful. For example, the NCD Alliance is a huge international alliance for the prevention of NCDs. It was founded in 2009 by the International Diabetes Federation (IDF) CEO Ann Keeling [37]. This alliance currently unites over 2000 civil society organizations who have implemented over 100 community interventions. One of their core functions is knowledge exchange. For that matter they published in 2017 the “NCD Civil Society Atlas” [38]. In this document they analyzed 38 initiatives focusing on key success factors so other institutions or professionals could learn and replicate the model.

This alliance started with international institutions who knew that partnerships and collaborations were vital to increase the reach and impact of your actions. The model has been replicated in over 170 countries. Our model is based on this one, following the successful processes that influenced their development [31].

Another example is OPEN (Obesity Prevention through European Network) [39]. OPEN is a European project, in this case built around one specific health problem. It started in 2014 with the intention of disseminating their methodology (EPODE) [40] between several partners focusing on locally based community interventions for the prevention of obesity in adolescents from deprived areas. Our collaborative platform´s community actions were inspired by the Spanish Health Ministry’s National Prevention and Health Promotion Strategy and has a lot in common with this methodology (local inter-sectorial structures that implement health promotion actions). In addition, our platform has a much more robust structure and does not limit itself to one specific health problem. With three working groups in the three levels of intervention, we can work with different risk factors and health determinants at the same time, believing that this will increase the effectiveness of the interventions.

On the other side, in the Spanish context, the Spanish Network of Healthy Universities (REUS) [41] has been working for over 10 years to make the university context a health promoter setting, implementing different community interventions especially those that promote physical activity and healthy eating. In this matter, we see the network not only as a great potential partner but also as a space of special interest [42].

Learning the lessons from all these experiences, we are making action plans between non-governmental institutions to work on achieving efficient community health interventions. This collaborative platform includes different types of community interventions, like political incidence interventions, health education interventions and interventions that change the context and conditions in which people live. We think that the functional structure of the platform allows us to focus our actions with different approaches on different levels, achieving deep changes in social determinants of health, that are at the end the biggest influencers over NCDs.

Recent national publications emphasize the importance of the creation of different coordination mechanisms that facilitate and improve multi-level and intersectoral community interventions [43]. They also express the great importance that community interventions are having on the different autonomous communities of Spain for the planning of primary health care services. Other pieces of international literature also support the importance and value of partnerships and collaboration in health promotion [44,45]. We think that our platform answers to the problem following evidence-based recommendations and could really be a game changer. Much more must be done to really tackle the NCD problem, but we think that this could be a great improvement and could play as a booster for local governments to work harder on the subject. Many challenges and opportunities have been faced in the process of launching the platform and will be faced in the future as expressed below.

### Main Challenges and Opportunities

In the 14 months that we have been working on the platform, our main challenges are similar to those found in the literature [15,46,47].

- Complexity and variability of the different contexts that the work is done, guaranteed long-time sustainability of partnerships and resources limitation. Our platform allows great variability and flexibility of actions inside the general conceptual framework. It allows for the implementation of different kinds of community interventions depending on what the situation requires or the community needs.
- Institutional differences between partners (structural, resources, procedures) that generate very complicated legal and bureaucratic barriers. We have been very careful with the creation process, trying to make it very participative and everything has been done by consensus. Having the objectives in mind, a common agenda was made to make easier the interaction between partners.
- Reaching an agreement on the roles and responsibilities when defining the platform was also an important issue between partners. In our collaborative platform, every institution established from the beginning in a questionnaire their possibilities and resources (human resources, communication efforts, branding) for the platform. This was added to the terms of reference that we all approved.
- Management of economic resources. From the beginning each institution contributed professional or different resources, avoiding any money exchange between partners.

As the fundamental opportunity we can say that for these types of platforms to work, it is very important to have institutional support. From the beginning, the project was presented to the Health Ministry´s Sub-Directorate General of Health Promotion and they have been giving technical support through the whole process. Using the National Prevention and Health Promotion Strategy elaborated by them creates trust and synergies, which facilitates the sustainability and efficacy of the platform´s work.

## 5. Conclusions

After the process of creating a collaborative platform for the development of community interventions to prevent non-communicable diseases (NCD) we think that some lessons learned are very important in achieving proper functioning, sustainability and efficacy:

- Institutional support and recognition.
- Multi-component community interventions.
- Collaboration between different agents and local authorities.
- Planning based on community needs.
- Evidence-based actions.

Considering the great diversity of factors that interact and influence people´s health, different types of community interventions (changing the context, local regulations, health education, tool boxes and resources) are every day more useful and necessary to create the conditions in which people can live and have healthy choices as the easy choices. For these interventions to be effective, real and sustainable collaboration and coordination between different agents and sectors is necessary.

## Figures and Tables

**Table 1:** Founding partners of the platform, main field and type of actions on non-communicable diseases.

Institution	Main Field	Type of Actions
Spanish Association Against Cancer (AECC)	Cancer	Prevention, Research, Political Incidence, Communication
Spanish Heart Foundation (FEC)	Heart Disease	Prevention, Health Promotion, Communication
Spanish Red Cross	General Health	Health Promotion, Political Incidence, Healthcare
Spanish Society of Family and Community Medicine (semFYC)	Primary Healthcare	Health Promotion, Healthcare, Research
Spanish Federation of Community Nursing Associations (FAECAP)	Primary Healthcare	Health Promotion, Healthcare, Research
Spanish Diabetes Association (SED)	Diabetes	Health Promotion, Healthcare, Research

## Appendix A

**Table A1:** Phases, actors, objectives, methodology description of the whole initiative. What is done and what is going to be.

Phase	Actor	Objectives	Methodology Description	Done	Planned 2019	To be Planned
Organizational	AECC All partners	Creating a collaborative platform by articulating non-governmental institutions	Mapping and evaluation of potential partners.	X		
			Meetings with potential partners.	X		
			Defining an organizational and functional structure	X		
			Defining roles, responsibilities, resources, etc.	X		
			Signing Terms of Reference		X	
Functional	Work group 1	Influence decision-makers to make permanent decisions and changes that promote healthy habits.	Political Incidence		X	
		Create political structures and networks that support health promotion through community interventions.				
	Work group 2	Promote the implementation of the National Prevention and Health Promotion Strategy.	Community Interventions		X	
		Promote the local implementation of the strategy (community interventions).				
		Design and implement community interventions to promote healthy lifestyles (health education and changes in the context).				
	Work group 3	Design health promotion interventions through social marketing and communications.	Communication		X	
		Perform as a communications team for the platform.				
Evaluation	All partners and each work group	Evaluating the implementation process of the initiative.	Qualitative evaluation		X	
		Evaluating the political incidence actions.	Quantitative evaluation			X
		Evaluating the results of each community intervention.	Quantitative evaluation			X
		Evaluating health promotion interventions through communications.	Quantitative evaluation			X
